# Human Papillomavirus Infection (HPV) Prevalence in the Black Sea Region of Turkey: Primary HPV Screening for Cervical Cancer

**DOI:** 10.7759/cureus.52615

**Published:** 2024-01-20

**Authors:** Mustafa Cengiz Dura, Hilal Aktürk, Özgür Aslan, Şükrü Yıldız, Mehmet Kefeli, Handan Çelik

**Affiliations:** 1 Obstetrics and Gynaecology, Bakırköy Sadi Konuk Education and Research Hospital, İstanbul, TUR; 2 Obstetrics and Gynaecology, Bakırköy Sadi Konuk Education and Research Hospital, Istanbul, TUR; 3 Department of Pathology, Faculty of Medicine, Ondokuzmayıs University, Samsun, TUR; 4 Obstetrics and Gynecology, Faculty of Medicine, Ondokuzmayıs University, Samsun, TUR

**Keywords:** cancer, cervical, co-test, smear, prevalence, hpv

## Abstract

Objective: Planning vaccination and treatment options requires knowledge about the regional incidence of human papillomavirus infection (HPV) and its genotypes. The aim of our study was to determine the regional prevalence of HPV with genotypic subclassification and to evaluate the efficacy of HPV testing in cervical screening.

Material and Method: This retrospective cohort study analyzed records of 10,152 women aged 30-65 from the On Dokuz Mayıs University Medical Faculty's Gynecology Clinic, excluding those with a history of cervical disease, hysterectomy, or current pregnancy. Pre- and postmenopausal and total HPV prevalence were calculated. There was a total of 544 patients who underwent a colposcopic biopsy after cervical screening. The research focused on comparing the efficacy of Pap smears, HPV tests, and co-tests in detecting LSIL or more severe conditions, utilizing the BD Viper LT System for HPV screening and liquid-based cytology for smear tests.

Results: The prevalence of HPV in our region was determined to be 10.9%. When considering menopausal status, HPV prevalence was found to be 9.8% in premenopausal individuals and 12.4% in postmenopausal individuals. Evaluation of the pap smear results revealed a sensitivity of 74.8% for premenopausal and 81% for postmenopausal patients, with a specificity of 51% observed in both menopausal categories. In contrast, HPV testing demonstrated a sensitivity of 90.8% in premenopausal and 92.4% in postmenopausal individuals, with a specificity of 58% for both groups. The co-test results indicated an even higher sensitivity, with 97.9% in premenopausal and 100% in postmenopausal individuals, albeit with a reduced specificity of 28% in both cases. When identifying LSIL (low-grade squamous intraepithelial lesions) and more severe conditions, the sensitivity and specificity of the primary HPV test surpassed those of the pap smear. While the primary HPV test's sensitivity is markedly lower compared to the co-test, it boasts a significantly higher specificity.

Conclusion: Regional HPV prevalence studies are valuable for the implementation of screening policies. The primary HPV DNA test is a reliable method for detecting preinvasive and invasive lesions in patients over 30 years of age.

## Introduction

High-risk or oncogenic human papilloma virus (HPV) types are agents that cause anogenital and oropharyngeal cancers in women, and HPV is estimated to be responsible for 5.2% of cancers worldwide [1]. According to the United States National Program of Cancer Registries, cervical cancer is ranked as the third most prevalent gynecological cancer [2]. In low-income countries with limited healthcare resources, it ranks as the second most prevalent cause of cancer-related deaths among women. In terms of both new cases and deaths, it rates 12th among cancers diagnosed in women in Turkey in 2020 [3]. The fact that cervical cancer is less common in Turkey compared to other countries has been attributed to the inadequacy of the notification system and access to health services [4]. 

Various studies conducted in Turkey found the prevalence of HPV between 2.1% and 25.7% [5]. The link between HPV infection and cervical cancer, which has a single factor contributing to its etiology, is unlike any other cancer type in that it has been demonstrated with such clarity [6]. Among HPV-related cancers, with the practice of detecting HPV DNA by polymerase chain reaction (PCR), HPV deoxyribonucleic acid (DNA) was detected in all patients with cervical cancer; all cases are associated with persistent HPV infection [5]. Because of this relationship, the importance of HPVDNA testing in preventing cervical cancer is clear.

Understanding the prevalence of human papillomavirus (HPV) and its various types within a specific population is crucial for devising effective vaccination and treatment plans. [7]. In our region, no HPV prevalence study has been carried out before. Studies on HPV prevalence in Turkey vary [8-10].

The objective of the present study is to retrospectively ascertain the prevalence of human papillomavirus (HPV) and its types in patients who were admitted to the gynecology polyclinic at a university hospital, which is the largest healthcare facility in the Black Sea region. Additionally, the study aims to compare the effectiveness of cytology, HPV DNA testing, and co-testing in premenopausal and postmenopausal patients.

## Materials and methods

This retrospective cohort study was carried out upon ethical approval in accordance with the decree (date: 23.03.2017, number: 129) of the Clinical Research Ethics Committee of On Dokuz Mayıs University Medical Faculty.

**Figure 1:**
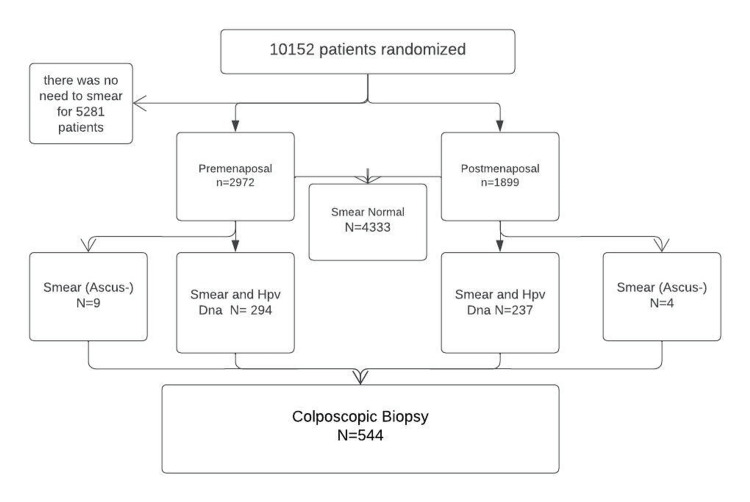
Flowchart on Methodology

This study was carried out through the retrospective examination of the records of 10152 patients admitted to the Gynecology Polyclinic of the Outpatient Clinic of Obstetrics and Gynecology at the University Medical Faculty between February 2015 and November 2017. Patients aged 30-65 years without a history of cervical invasive disease were included in the study. A retrospective review of the records was carried out through the hospital automation system. Patients who had undergone hysterectomy for any reason, patients with a history of cervical invasive disease, and patients with a current pregnancy were excluded from the study. Patients who received the HPV vaccine at any time in their lives were not included in the study (Figure 1).

HPV prevalence was calculated in both pre- and postmenopausal groups, and colposcopic biopsy results were analyzed. Patients whose menstrual periods were determined to have been permanently interrupted retrospectively for 12 months without a pathological or physiological reason were considered to be menopausal. A total of 544 patients who underwent colposcopic biopsy were identified in the scanned patient group. In order to compare the effectiveness of the tests, sensitivity, specificity, positive predictive value, and negative predictive value were calculated according to smear, HPV test, and co-test results for low-grade squamous intraepithelial lesion (LSIL) (CIN1) and above lesions. 

In the laboratory of the research center, the BD Viper LT System is used for HPV screening. This system works with a real-time PCR assay for the qualitative detection of 14 high-risk HPV types. The BD Onclarity HPV Assay detects HPV genotypes 16, 18, 45, 31, 51, 52, 33/58, 35/39/68, and 56/59/66. For the smear test, the liquid-based thin layer cytology method is used. A sample taken with a Cytobrush is placed in a liquid fixative solution; after centrifugation, the cells in the liquid are compressed onto a filter and then coated by a single-layer glass slide in the liquid cytology laboratory. The HPV test can be studied from the same sample. In preparation for a colposcopic biopsy, the vagina and cervix are cleaned with sterile distilled water, and the cervix is enlarged and examined with the naked eye. Following the procedure, 3% acetic acid is applied to the cervix, and the green filter feature is used to better visualize atypical vasculature. A biopsy is taken with biopsy forceps from areas observed to be abnormal. Then, a repeat biopsy is taken from the areas observed to be abnormal following the application of the lugol solution. Colposcopy procedures were performed by certified specialists.

The statistical techniques employed for the majority of the analysis were the Chi-square method and Fisher's exact test. Given the non-normal distribution of age in relation to HPV positivity, the Mann-Whitney U test was employed. All statistical tests were conducted using a two-tailed significance level of 5%. The statistical analysis was conducted utilizing SPSS 17 (Statistical Package for Social Sciences, Chicago, US).

## Results

In the study, HPV prevalence was found to be 10.9%. Premenopausal patients are described as those experiencing irregular menstruation and spotting between periods. Postmenopausal patients are defined as individuals who have not had a menstrual period in the last year and may also exhibit symptoms associated with menopause, such as palpitations and hot flashes. The ages of the patients ranged from 30 to 65 years, with a mean age of 44.5 ± 8.90 years. The age range of premenopausal patients was between 30 and 50, and their mean age was 38.0 ± 4.90 years, while the age range of postmenopausal patients was between 40 and 65, and their mean age was 52.0 ± 5.70 years. The prevalence of HPV in premenopausal and postmenopausal patients in our region is shown in Table 1. 

**Table 1:** Prevalence of total HPV in premenopausal and postmenopausal patients

	Premenopausal (n= 2972)	Postmenopausal (n = 1899)	TOTAL (n = 4871)
HPV16	116 (3.9%)	135 (7.1%)	5.1% (n = 251)
HPV18	26 (0.8%)	14 (0.7%)	0.8% (n = 40)
Other high-risk HPV	140 (4.7%)	85 (4.4%)	4.6% (n = 225)
HPV16+Other high-risk HPV	12 (0.4%)	3 (0.1%)	0.3% (n=15)
TOTAL	294 (9.8%)	237 (12.4%)	10.9%

To compare the effectiveness of the tests, it was determined that colposcopic biopsy was performed in 544 of 10152 patients who underwent HPV or smear testing by retrospective archive scanning of the samples. 

Colposcopic biopsy results of 544 patients revealed 67.5% (n = 367) benign, 32.5% (n = 177) LSIL (CIN1), and higher lesions. The rate of benign biopsy results was 67.5% (n = 367). LSIL (CIN1) rate was 10.8% (n = 59), (HSIL) CIN2 rate was 4.8% (n = 26), HSIL (CIN3) rate was 12.5% (n = 68), squamous cell carcinoma rate was 2.9% (n = 16), and endocervical adenocarcinoma (endocervical carcinoma in situ (AIS) and invasive endocervical adenocarcinoma) rate was 1.5% (n = 8). Colposcopic biopsy results are summarized in Table 2.

**Table 2:** Results of a colposcopic biopsy LSIL: low-grade squamous intraepithelial lesions, HSIL: High-grade squamous intraepithelial lesions

Biopsy Result	Number	Percentage
LSIL (CIN 1)	59	10.8%
HSIL (CIN 2)	26	4.8%
HSIL (CIN 3)	68	12.5%
Squamous cell carcinoma	16	2.9%
Endocervical adenocarcinoma	8	1.5%
Total	177	32.5%

The HPV genotype distribution in premenopausal and postmenopausal patients who underwent a colposcopic biopsy is summarized in Table 3.

**Table 3:** HPV genotype distribution in patients undergoing colposcopic biopsy. HR: High risk

	Premenopausal (n = 178)	Postmenopausal (n = 136)	Total (n = 314)
HPV16	94 (29.7%)	70 (30.7%)	164 (52%)
HPV18	10 (3.2%)	5 (2.2%)	15 (4.7%)
Other HPV HR	64 (20.3%)	56 (24.6%)	120 (38.2%)
HPV16 + HR HPV positivity	10 (3.2%)	5 (2.2%)	15 (4.7%)

Of the 544 patients who underwent colposcopic biopsy, 42% (n = 230) were patients who had a negative HPV test result but had to undergo biopsy due to a smear abnormality. In this group, the total HPV prevalence was calculated at 58%. Of the 314 patients with a positive HPV test result, 52% (n = 164) had HPV16 positivity, 4.7% (n = 15) had HPV18 positivity, 38.2% (n =120) had high-risk HPV positivity, and 4.7% (n=15) had HPV16 + high-risk HPV positivity. The evaluation of these results reveals that the distribution of HPV types is similar in premenopausal and postmenopausal patients who underwent colposcopic biopsy.

Colposcopic biopsy results of premenopausal and postmenopausal patients concerning smear, HPV, and co-test results are listed in Table 4, and drawing upon these results, the sensitivity, specificity, positive predictive value, and negative predictive value of premenopausal and postmenopausal patients are calculated in Table 5.

**Table 4:** Evaluation of colposcopic biopsy results of patients according to smear, HPV test, and co-test results

Menopausal Status	Smear test	Benign	LSIL (CIN1) and higher lesions
Premenopausal	Abnormal	106 (56.4%)	82 (43.6%)
	Normal	112 (87.5%)	16 (12.5%)
Postmenopausal	Abnormal	73 (53.3%)	64 (46.7%)
	Normal	76 (83.5%)	15 (16.5%)
Menopausal Status	HPV test	Benign	LSIL (CIN1) and higher lesions
Premenopausal	Positive	91 (50,6%)	89 (49,4%)
	Negative	127 (93,4%)	9 (6,6%)
Postmenopausal	Positive	62 (45,9%)	73 (54,1%)
	Negative	87 (93,5%)	6 (6,5)
Menopausal Status	Co-test	Benign	LSIL (CIN1) and higher lesions
Premenopausal	Negative	174 (88.3%)	23 (11.7%)
	Positive	44 (37%)	75 (63%)
Postmenopausal	Negative	121 (85.2%)	21 (14.8%)
	Positive	28 (32.6%)	58 (67.4%)

**Table 5:** Calculation of sensitivity, specificity, PPV, and NPV according to test results PPV: Positive predictive value , NPV: Negative predictive value

Smear Test	Premenopausal	Postmenopausal
Sensitivity	74,8 %	81,0 %
Specificity	51,4 %	51,0 %
PPV	43,6 %	46,7%
NPV	87,5 %	83,5 %
HPV Test	Premenopausal	Postmenopausal
Sensitivity	90,8 %	92,4%
Specificity	58,3 %	58,4 %
PPV	49,4%	54,1 %
NPV	93,4 %	93,6 %
Co-test	Premenopausal	Postmenopausal
Sensitivity	97,9 %	100 %
Specificity	28,8 %	28,2 %
PPV	38,6 %	42,5 %
NPV	97,0 %	100 %

The sensitivity of the smear results in detecting LSIL (CIN1) and higher lesions was 74.8% for premenopausal patients and 81% for postmenopausal patients. Specificity was 51% for both menopausal conditions. Positive predictive value (PPV) was 43.6% in premenopausal and 46.7% in postmenopausal patients. Negative Predictive Value (NPV) was 87.5% in premenopausal patients and 83.5% in postmenopausal patients (Table 5).

It was shown that the HPV test had a sensitivity of 90.8% in premenopausal patients and a sensitivity of 92.4% in postmenopausal patients. Based on both types of menopause, the specificity value was found to be 58%. In premenopausal patients, the PPD value was 49.4%, whereas it was 54.1% in postmenopausal patients. While the NPD value was calculated to be 93.4% premenopausal, it was calculated to be 93.6% postmenopausal. Consequently, there was no notable dissimilarity in any variable between postmenopausal and premenopausal values, as per statistical analysis (Table 5).

The sensitivity and negative predictive value of the HPV test were significantly higher than those of the smear test.

In the co-test between pre- and postmenopausal patients, no significant difference was observed in specificity, sensitivity, PPV, or NPV values. When a co-test was used, its sensitivity was higher than that of the HPV test, but its specificity was significantly lower.

The sensitivity and specificity of the primary HPV test were higher than those of the smear test in the detection of LSIL and higher lesions. Compared to the co-test, the sensitivity of the primary HPV test is lower, but its specificity is significantly higher.

## Discussion

In the prevalence studies conducted in Western countries, the prevalence of HPV is reported to be higher than in our study and other studies conducted in Turkey [11]. The fact that sexual life in our country is more traditional compared to western countries, as well as a lower number of partners, may be one of the reasons for the difference between our group and the literature. The fact that almost all men in Turkey are circumcised can also be a protective factor [12,13]. In Spain, Castellsaque et al. [14] suggested that circumcision not only reduces HPV contamination and transmission but also reduces the likelihood of cervical cancer in the female partner. Similarly, Hernandez et al. and Auvert et al. reported that the prevalence of HPV is higher in uncircumcised men, and circumcision is protective against cervical cancer [15,16]. In our study of oncogenic HPV genotypes in our region, we obtained results similar to those of genotype studies in Turkey and worldwide [16-18]. Furthermore, our study did not find any statistically significant disparity in the prevalence of oncogenic HPV genotypes between premenopausal and postmenopausal patients.

The relationship between cervical cancer, HPV, and preinvasive cervical intraepithelial neoplasia is well known. HPV leads to preinvasive cervical lesions, which, if not recognized and treated early, lead to invasive cervical lesions. In this study, six of the seven detected cases of squamous cell carcinoma had HPV16 positivity, and one had high-risk HPV positivity. HPV16 positivity was detected in two cases of carcinoma in situ. Among the four cases with endocervical adenocarcinoma, one had HPV16, one had HPV18, and two were HPV-negative. 

Our study revealed that all efficacy parameters of the primary HPV test and co-test were higher than those of cytology. When the primary HPV test and co-test were compared, the sensitivity and NPV of the co-test were significantly higher than those of the primary HPV test. However, the specificity and PPV of the co-test were significantly lower. In a study published in 2015, Blatt et al. [19] reported that, for the detection of HSIL (CIN 3) and higher lesions, screening with a co-test in women aged 30-65 years was more sensitive than screening by only HPV and only cytology (sensitivity rates of 98.8%, 94%, and 91.3%, respectively). The specificity of the positive cytology was calculated at 26.3%, the specificity of the positive HPV DNA was 25.6%, and the specificity of the positive co-test was calculated at 10.9% [20]. Similar results were obtained in our study. Co-test sensitivity in the same age group was significantly higher in both groups than the HPV test and cytology.

According to the results of the ATHENA study, which is the first prospective study on primary HPV screening, primary HPV screening for the detection of HSIL (CIN3) and higher lesions in women over the age of 25 was reported to have higher sensitivity than screening by only cytology and co-testing (cytology below 30 years of age, co-test above 30 years) [21]. The Athena study is the most comprehensive prospective study on the HPVDNA test in the United States as a primary test for screening cervical cancer. In this study, it was reported that the primary HPVDNA test was more sensitive in detecting HSIL+ (CIN3) lesions compared to cytology and the co-test in women over 25 years of age [22].

According to research findings, the utilization of primary HPV DNA testing for screening purposes is more sensitive but less specific compared to cytology in the detection of cervical preinvasive lesions [23]. While high sensitivity makes it possible to perform screening at shorter intervals than cytology, low specificity leads to a large number of patients being referred for a colposcopy [24]. Compared to the co-test, the sensitivity of the primary HPV test is lower, but its specificity is significantly higher. In light of the data obtained in our study, it is appropriate to use the HPVDNA test as a primary screening method for cervical cancer screening in patients over 30 years of age. Similar to the previous research findings, we found that the primary HPV test had lower sensitivity but much higher specificity than the co-test. As we mentioned above, low specificity will increase the rate of excess, unnecessary colposcopy. 

The strength of the study is that there was no prevalence study in our region before. The limitation is that the efficiency of the tests is compared based on LSIL (CIN1) biopsy results. Biomarker studies performed and to be performed with the aim of maximizing the efficacy of primary HPV screening with maximum sensitivity and minimum false-positive results would be guiding [25]. 

In the process of cervical cancer screening and diagnosis, risk stratification is conducted based on the pathology findings post-colposcopy, following initial assessments via smear tests and HPV DNA testing. It is posited that artificial intelligence may offer further enhancements to this paradigm in the future [26].

## Conclusions

Cervical cancer can be prevented with screening tests. The utilization of HPV DNA testing is a crucial component of screening initiatives in Turkey. The utilization of the HPVDNA test as the primary screening method for cervical cancer screening in patients over 30 years of age is a dependable approach for detecting preinvasive and invasive lesions.
